# Antiplatelet drugs and the perioperative period: What every urologist needs to know

**DOI:** 10.4103/0970-1591.56174

**Published:** 2009

**Authors:** Pawan Vasudeva, Apul Goel, Vengetesh K. Sengottayan, Satyanarayan Sankhwar, Divakar Dalela

**Affiliations:** Department of Urology, C.S.M.M.U (Upgraded King George Medical College), Lucknow, Uttar Pradesh, India

**Keywords:** Antiplatelet drugs, aspirin, hemorrhage, perioperative period, thrombosis, TURP

## Abstract

Antiplatelet agents like aspirin and clopidogrel are widely used for indications ranging from primary and secondary prevention of myocardial infarction or stroke to prevention of coronary stent thrombosis after percutaneous coronary interventions. When patients receiving antiplatelet drugs are scheduled for surgery, urologists commonly advise routine periprocedural withdrawal of these drugs to decrease the hemorrhagic risks that may be associated if such therapy is continued in the perioperative period. This approach may be inappropriate as stopping antiplatelet drugs often exposes the patient to a more serious risk, i.e. the risk of developing an arterial thrombosis with its potentially fatal consequences. Moreover, it has been seen that the increase in perioperative bleeding if such drugs are continued is usually of a quantitative nature and does not shift the bleeding complication to a higher risk quality. We, in this mini review, look at the physiological role and pathological implications of platelets, commonly used antiplatelet therapy and how continuation or discontinuation of such therapy in the perioperative period affects the hemorrhagic and thrombotic risks, respectively. Literature on the subject between 1985 and 2008 is reviewed. The consensus that seems to have emerged is that the policy of routine discontinuation of antiplatelet drugs in the perioperative period must be discouraged and risk stratification must be employed while making decisions regarding continuation or temporary discontinuation of antiplatelet therapy. Although antiplatelet drugs may be discontinued in patients at a low risk for an arterial thrombotic event, they must be continued in patients where the risks of bleeding and complications related to excessive bleeding are less than the risks of developing arterial thrombosis.

## INTRODUCTION

Antiplatelet drugs are now widely prescribed, with upto 25% of the elderly population receiving aspirin.[1] When patients receiving antiplatelet therapy are scheduled for surgery, e.g. for transurethral surgery, urologists commonly advise temporary discontinuation of antiplatelet drugs starting at least 10 days before surgery.[2] The rationale is that there is a potential risk of increased bleeding if such therapy is continued in the perioperative period. In a survey conducted in the United Kingdom involving 287 urologists, 62% asked their patients to stop aspirin before a transurethral resection of the prostate (TURP) and among these 178 urologists, 62 % stopped aspirin in all patients regardless of indication.[3] The often-ignored flip side of routine perioperative antiplatelet drug discontinuation is that it exposes the patient to the risk of developing arterial thrombosis, which may have potentially fatal consequences.[4] This thrombotic risk may be even more relevant in urological patients, many of whom are elderly and have associated comorbidities. In a series, 50% of the men undergoing prostate surgery had concurrent hypertension and 20% had ischemic heart disease.[5] In this article, we review the physiological function and pathological role of platelets, the commonly used antiplatelet drugs and the evidence regarding benefits and risks of continuing/discontinuing antiplatelet therapy perioperatively. Evidence-based perioperative management of patients on antiplatelet drugs (i.e. whether to stop/change or continue antiplatelet therapy in such patients) is presented based on recently published guidelines. This mini review is based on articles searched from 1985 to 2008 on “Pubmed” and “Google” using key words antiplatelet drugs, aspirin, TURP, hemorrhage, thrombosis and perioperative period.

## PLATELETS: PHYSIOLOGICAL ROLE AND PATHOLOGICAL IMPLICATIONS

Platelets are anucleate cells produced from megakaryocytes in the bone marrow and their primary function is to stop hemorrhage after tissue trauma and vascular injury. The physiological response at the site of vascular injury is complex and, among others, involves platelet activation, adhesion and release of various autocrine and paracrine mediators like thromboxane A2, epinephrine, adenosine diphosphate (ADP), etc. These mediators then amplify the platelet response, resulting in recruitment of more platelets from circulation and platelet aggregation leading to the formation of the haemostatic plug.[6]

Unfortunately, platelets are also implicated as key cellular elements in many pathological derangements of the vascular system. By virtue of their capacity to adhere to sites of vascular injury, platelets play an important role in the development of potentially life-threatening acute coronary syndromes and cerebrovascular events, where the usual mechanism leading to the acute episode is atherosclerotic plaque disruption (which may be considered a form of vessel injury) followed by platelet activation and thrombosis.[7–10] Platelets, by virtue of being a source of inflammatory mediators, are also thought to participate in the process of atherosclerosis, which itself is considered a chronic inflammatory disease. Animal studies have shown that platelet activation increases the rate of atherosclerotic plaque formation[11] and that inhibition of the synthesis of platelet thromboxane delays atherogenesis.[12] The evidence of the role of platelets in the development of chronic atherosclerotic lesions in humans, although, is largely indirect.[13]

In an attempt to address this central role of platelets in atherothrombosis, antiplatelet drugs are prescribed by physicians and there is ample clinical evidence to support their use in a variety of clinical situations where the patient is at risk of arterial thrombi.[14,15] Commonly, such therapy is prescribed for primary or secondary prevention of myocardial infarction or stroke, for prevention of coronary stent thrombosis after percutaneous coronary intervention (PCI), in peripheral arterial occlusive disease, etc.[16–18] In the primary prevention setting, a metaanalysis has shown that aspirin therapy results in 32% relative risk reduction for myocardial infarction and a 15% relative risk reduction for all vascular events.[19,20] In the secondary prevention setting, antiplatelet therapy reduces the incidence of non-fatal myocardial infarction by one-third, non-fatal stroke by one-quarter and vascular mortality by one-sixth.[15]

## ANTIPLATELET DRUGS IN CLINICAL USE

Aspirin, the most popular drug in this class, exerts its antiplatelet action by acetylating and irreversibly inactivating the cyclooxygenase 1 (COX1) enzyme, which results in blocking the pathway of thromboxane A2 synthesis. With the platelet unable to synthesize thromboxane A2, a potent platelet aggregator and vasoconstrictor, the prostaglandin biosynthetic pathway, loses its capability of increasing platelet aggregation. The onset of action of aspirin is rapid, approximately 30 min, and the antiplatelet effect is irreversible, i.e. it will persist for the life of the platelet (mean life span being 8–10 days in circulation). Because of their anucleate nature, platelets are incapable of synthesizing new enzyme to replace the inactivated one. Restoration of normal aggregation after aspirin therapy cessation is caused by young newly released non-acetylated platelets replacing the aspirin-treated ones.[21]

The concept of low-dose aspirin in cardiology stems from the differential effect that the drug has on the two variants of the COX enzyme, with the effect being nearly 100-fold more for the COX1 variant. At low doses, COX1 is selectively inhibited and hence only thromboxane A2 production is affected. When higher and more frequent dosing is employed, decrease in PGI2 synthesis consequent to inhibition of COX2 pathway also occurs, which in fact may be detrimental to the antithrombotic effect of aspirin because PGI2 itself is an inhibitor of platelet aggregation. Hence, in the setting where aspirin is prescribed for its antiplatelet effect, use of the lowest effective dose (50–100 mg/day for long-term treatment) is currently considered the most appropriate strategy.[22]

Clopidogrel, another commonly used antiplatelet drug, is an ADP-receptor antagonist. It irreversibly inhibits the low-affinity ADP receptor, P2Y12, on the platelet membrane resulting in selective inhibition of ADP-induced platelet aggregation.[23] Clopidogrel is usually started at a dose of 75 mg/day. With this dose, it takes 3–5 days for steady state levels to be reached. Another option is to start with a loading dose of 300–600 mg and then shift to 75 mg/day. In this way, platelet inhibition effect is demonstrable within a few hours. Like in the case of aspirin, platelet studies return to normal after about 1 week of stopping therapy as a result of the influx of new untreated platelets.[24]

Ticlopidine, another drug that belongs to the same family as clopidogrel, has now been virtually abandoned because of the frequency of side effects, which include neutropenia (1–2.4%) and thrombotic thrombocytopenic purpura (1 in 3000).[21]

Glycoprotein IIb-IIIa antagonists are novel drugs (Abciximab, Tirofiban, Eptifibatide) that act on the Glycoprotein IIb-IIIa binding site on the platelet membrane and interfere with the final common pathway of platelet aggregation. These drugs are used for the prevention of immediate thrombosis of coronary stents and are prescribed for 24–48 h after PCI.[21]

## HEMORRHAGIC RISKS IF ANTIPLATELET DRUGS ARE CONTINUED PERIOPERATIVELY

The published literature on this subject in so far as urologic procedures are concerned largely involves studies that have assessed bleeding risks in aspirin users who have undergone either TURP or transrectal prostatic biopsy.

Following reports of two deaths in the early 1990s' in aspirin users after prostatectomy due to increased bleeding, studies were undertaken to address the effect of low-dose aspirin on bleeding after TURP. We identified four such studies. Two studies reported increased hemorrhagic risks, with one documenting a relationship between severe hemorrhage and aspirin ingestion[25] and the other reporting a significantly higher transfusion requirement among aspirin users.[26] In another study, the authors did not find any difference in the mean blood loss among 40 aspirin users and 42 control patients.[27] In the fourth and the only prospective, randomized, placebo-controlled study evaluating the effect of low-dose aspirin on bleeding after TURP, the authors did not find any significant difference in the operative blood loss or in the transfusion requirements, although post-operative blood loss was significantly increased in the aspirin group.[28] Another recent study reported that early aspirin initiation (24 h after discontinuation of bladder irrigation) was not associated with increased post-operative bleeding when compared with late (3weeks after surgery) aspirin initiation after transurethral prostate or bladder surgery.[29]

Among four studies identified, which have addressed bleeding risks in aspirin users undergoing transrectal prostatic biopsy, two studies did not find any increased post-procedural bleeding among aspirin users.[30,31] In one study, although the incidence of mild bleeding complications was not higher, the duration of self-limiting hematuria and rectal bleeding was prolonged.[32] In the fourth study the authors reported a higher cumulative incidence of hematuria and rectal bleeding but not of hematospermia among aspirin users. Further, aspirin users had a longer mean duration of bleeding, although there was no increase in bleeding severity.[33]

In a metaanalysis involving 41 studies (12 observational retrospective, 19 observational prospective, 10 randomized), which reported on 49,590 patients (14,981 on aspirin) undergoing a variety of non-cardiac surgeries, it was found that although periprocedural aspirin increased the rate of bleeding complication by a factor of 1.5, it did not lead to a higher level of severity of bleeding complications (with the exception of intracranial surgery and possibly transurethral prostatectomy). The analysis also revealed that aspirin increased the risk of transfusion after transurethral prostatectomy by a factor of 2.7, i.e. whereas aspirin users received on an average 0.4–5.0 units of red blood cells, the average for the control patients was much less, at 0.3–1.7 units.[34]

In summary, it may be fair to say that although aspirin increases the frequency of bleeding complication in the perioperative period, the increase, mostly, is only quantitative and does not move the bleeding complication toward a higher risk quality.[34]

## THROMBOTIC RISKS OF DISCONTINUING ANTIPLATELET DRUGS PERIOPERATIVELY

The systemic inflammatory syndrome and the acute phase reaction associated with surgery are known to increase platelet adhesiveness and decrease fibrinolysis, resulting in a prothrombotic state.[35,36] Hence, antiplatelet drug therapy continuation may be considered even more important in patients at risk of arterial thrombosis during the perioperative period. If such therapy is discontinued abruptly, not only is the protective effect lost but a rebound phenomenon associated with drug withdrawal also results, which accentuates the problem, e.g. abrupt withdrawal of aspirin itself results in abnormally high levels of thromboxane A2 and a prothrombotic state.[37] There are reports of myocardial infarction and death, transient ischemic attacks, cerebrovascular accident and death, etc. reported in aspirin users undergoing TURP and who had stopped aspirin perioperatively.[4] Although the exact incidence of atherothrombosis following aspirin withdrawal is not known, cessation of such therapy precedes up to 10.2% of acute cardiovascular syndromes. In a metaanalysis, the time interval between aspirin discontinuation and acute cerebral events, acute coronary syndromes and acute peripheral arterial syndromes was reported to be 14.3 ± 11.3, 8.5 ± 3.6 and 25.8 ± 18.1 days, respectively.[34]

The risk of hazardous events like stroke, myocardial infarction, etc. to which a patient would be exposed also largely depends on the clinical indication for which antiplatelet therapy was started in the first place. In one study, six of seven patients undergoing non-cardiac surgery within 3 weeks of having undergone coronary stenting (which is considered a high-risk clinical situation for thrombosis) in whom thienopyridine antiplatelet therapy was stopped died as compared with one in 20 in whom the drug was continued, the death being ascribed to stent thrombosis in all patients who did die.[38]

To summarize, because the risks of withdrawing antiplatelet agents in the perioperative period may at times be higher than those of maintaining this vital medication, it may be inappropriate to advise routine discontinuation of antiplatelet drugs in the perioperative period.

One way of dealing with patients on antiplatelet therapy is to adopt a surgical approach/procedure that minimizes bleeding risks and allows for safe surgery even while these drugs are continued, e.g. laser prostatectomy in lieu of a TURP. Although no guidelines exist for perioperative management of antiplatelet therapy based on the nature of surgery, there is now literature available to guide clinical decisions largely based on the risk of cardiac event that the patient is prone to during the perioperative period.

It is in this regard that the American College of Chest Physicians (ACCP) has recently published evidence-based clinical practice guidelines for perioperative management of patients on antiplatelet therapy.[39]

## ACCP GUIDELINES [TABLE 1]

**Table 1:** Simplified American College of Chest Physicians guidelines (2008) on perioperative management of patients receiving antiplatelet therapy

Category	Preoperatively receiving	Recommendation for perioperative period
Not at a high risk for cardiac event (e.g., primary prevention setting)	Aspirin	Stop aspirin
At a high risk of cardiac event (e.g., within 3 months postmyocardial infarction)	Aspirin	Continue aspirin
	Aspirin and clopidogrel	Continue aspirin, stop clopidogrel
Special high-risk situation (within 6 weeks of bare metal coronary stent insertion/12 months of drug eluting coronary stent insertion)	Aspirin and clopidogrel	Continue aspirin, continue clopidogrel

1. For patients who are not at a high risk for cardiac events (typically, patients receiving aspirin for primary prevention of myocardial infarction/stroke), discontinuation of antiplatelet drugs is recommended [grade of recommendation (GOR):1C].
2. For patients at a high risk of cardiac events (e.g. patients who have suffered a myocardial infarction within the past 3 months, etc.) scheduled for non-cardiac surgery, continuation of aspirin up to and beyond the time of surgery is recommended (GOR: 2C). In addition, if such patients are receiving clopidogrel, discontinuation of the drug is recommended (GOR: 2C).
3. A special high-risk situation is patients with a bare metal stent (BMS)/drug eluting stent (DES) in the coronary arteries. In such patients, the thrombotic risk is very high if antiplatelet therapy is interrupted perioperatively, especially when surgery is undertaken in close proximity to the time of stent placement. Further, the clinical impact of thrombosis in this setting is considerable, as it would be fatal or associated with a large myocardial infarction in >50% of the affected patients. Although surgery should be avoided during the early period of endothelialization around the stent, patients requiring surgery within 6 weeks or 12 months of placement of BMS and DES respectively must be maintained on dual antiplatelet therapy, i.e. aspirin and clopidogrel during the perioperative period (GOR: 1C).
4. Patients in whom temporary drug interruption is indicated, the drug (aspirin/clopidogrel) should be stopped 7–10 days before the operative procedure and it may be resumed approximately 24 h after surgery when there is adequate hemostasis (GOR: 2C).

One unresolved issue is regarding the dose at which clopidogrel is to be resumed in patients who were receiving the drug pre-operatively. If therapy is resumed with 75 mg/day, it takes five or more days to achieve maximal platelet function inhibition. On the other hand, if therapy is resumed with a loading dose of 300 mg/day, although maximal platelet inhibition is seen within 2–15 h after administration, this increased dose also increases the hemorrhagic risk. Ultimately, dose of resumption will depend on the perceived risk of thrombosis, which would be reflected from other parameters like whether a patient has a coronary stent, the type of stent implanted and how recently was the stent implanted.

Patients in whom antiplatelet therapy is continued during the perioperative period, there is no need to routinely use platelet function assays to monitor the antithrombotic effect of aspirin or clopidogrel (GOR: 2C).

## MANAGING SEVERE PERIOPERATIVE HEMORRHAGE IN PATIENTS IN WHOM ANTIPLATELET THERAPY WAS CONTINUED

If a patient in whom antiplatelet therapy was continued has severe hemorrhage perioperatively, platelet transfusion is the only therapeutic option that can be envisaged to limit bleeding even though there are no clinical studies validating the benefits of platelet transfusion in limiting bleeding when it occurs (therapeutic administration) in patients with drug-induced thrombopathy.[40] One issue to consider before transfusing platelets in an emergency situation is the timing of the last clopidogrel dose. Because the half-life of clopidogrel is 4 h, its plasma level reaches close to zero after about 12 h (three half-lives) and it is only after 6–8 h after the last dose of clopidogrel that the transfused platelets will not be significantly affected by the drug.[41]

## SUMMARY

In summary, it is prudent that the urologist incorporates risk stratification in decisions concerning temporary interruption or continuation of antiplatelet therapy. Antiplatelet drugs should only be discontinued perioperatively if the known or assumed perioperative bleeding risks and their sequels are expected to be similar or more severe than the observed cardiovascular thrombotic risks after antiplatelet therapy withdrawal. This may require discussion of the merits of the case, often with a cardiology colleague.
